# Supplementary material for: Effect of surgical approach to hip arthroplasty on postoperative pain and mobilization and on efficacy of intrathecal oxytocin for pain
Source: medRxiv. 2025 Feb 26:2025.02.22.25322560. Preprint. [Version 1] doi: 10.1101/2025.02.22.25322560 (PMC11888492; doi:10.1101/2025.02.22.25322560)
Supplement: 1 [file NIHPP2025.02.22.25322560V1-supplement-1.pdf]

medRxiv preprint doi: https://doi.org/10.1101/2025.02.22.25322560; this version posted February 26, 2025. The copyright holder for this preprint (which was not certified by peer review) is the author/funder, who has granted medRxiv a license to display the preprint in perpetuity.
It is made available under a CC-BY 4.0 International license .

Supplementary Appendix 1: Spinal Oxytocin Hip Surgery Collaborators

Regina S. Curry, RN
Sean W. Dobson, MD
Christopher J. Edwards, MD
Daryl S. Henshaw, MD
J. Douglas Jaffe, MD
James D. Turner, MD
Robert S. Weller, MD

All from Department of Anesthesiology, Wake Forest University School of Medicine, Winston-Salem, NC

medRxiv preprint doi: https://doi.org/10.1101/2025.02.22.25322560; this version posted February 26, 2025. The copyright holder for this preprint (which was not certified by peer review) is the author/funder, who has granted medRxiv a license to display the preprint in perpetuity.
It is made available under a CC-BY 4.0 International license .

385

Supplementary Table 1. Post Hoc Sensitivity Analysis

	Outcome Intercept			Trajectory (Slope) of Outcome Over Time[a]		
	**Mean Difference**	**95% Confidence Interval**	**p**	**Mean Difference**	**95% Confidence Interval**	**p**
**Postoperative Steps**						
Posterior Approach	549.18	-808.35, 1906.71	0.43	-318.32	-548.98, -87.66	**0.007**
Oxytocin	-98.50	-1284.66, 1087.66	0.87	70.19	-133.56, 273.94	0.50
Posterior Approach x Oxytocin	-706.03	-2607.45, 1195.38	0.47	649.24	323.73, 974.75	**<0.001**
Baseline Steps	-0.07	-0.20, 0.07	0.35	0.17	0.15, 0.20	**<0.001**

Data from 85 patients was available for analysis of postoperative steps. Model was adjusted for age, sex, hospital site surgery was performed at, and mean baseline steps preoperatively. For evaluating trajectory over time, model was further included natural log of time (postoperative day) and a random intercept for each participant. [a]Values are reported for each variable times the natural log of the postoperative day after discharge. Thus, it represents changes in the slope of the outcome trajectory over time.
